# Whole genome analysis of hypervirulent *Klebsiella pneumoniae* isolates from community and hospital acquired bloodstream infection

**DOI:** 10.1186/s12866-017-1148-6

**Published:** 2018-01-08

**Authors:** Chaitra Shankar, Balaji Veeraraghavan, Laura Eve Buchnan Nabarro, Raji Ravi, Naveen Kumar Devanga Ragupathi, Priscilla Rupali

**Affiliations:** 1 Department of Clinical Microbiology, Christian Medical College, Vellore, Tamilnadu 632004 India; 2 Department of Infectious Diseases, Christian Medical College, Vellore, Tamil Nadu India

**Keywords:** Whole genome sequencing, Hypervirulent, *K. pneumoniae*, *K. quasipneumoniae*, Bacteremia, Virulence factors

## Abstract

**Background:**

Hypervirulent *K. pneumoniae* (hvKp) causes severe community acquired infections, predominantly in Asia. Though initially isolated from liver abscesses, they are now prevalent among invasive infections such as bacteraemia. There have been no studies reported till date on the prevalence and characterisation of hvKp in India. The objective of this study is to characterise the hypervirulent strains isolated from bacteraemic patients for determination of various virulence genes and resistance genes and also to investigate the difference between healthcare associated and community acquired hvKp with respect to clinical profile, antibiogram, clinical outcome and molecular epidemiology.

**Results:**

Seven isolates that were susceptible to all of the first and second line antimicrobials and phenotypically identified by positive string test were included in the study. They were then confirmed genotypically by presence of *rmpA* and *rmpA2* by PCR. Among the study isolates, four were from patients with healthcare associated infections; none were fatal. All patients with community acquired infection possessed chronic liver disease with fatal outcome. Genes encoding for siderophores such as aerobactin, enterobactin, yersiniabactin, allantoin metabolism and iron uptake were identified by whole genome sequencing. Five isolates belonged to K1 capsular type including one *K. quasipneumoniae*. None belonged to K2 capsular type. Four isolates belonged to the international clone ST23 among which three were health-care associated and possessed increased virulence genes. Two novel sequence types were identified in the study; *K. pneumoniae* belonging to ST2319 and *K. quasipneumoniae* belonging to ST2320. Seventh isolate belonged to ST420.

**Conclusion:**

This is the first report on whole genome analysis of hypervirulent *K. pneumoniae* from India. The novel sequence types described in this study indicate that these strains are evolving and hvKp is now spread across various clonal types. Studies to monitor the prevalence of hvKp is needed since there is a potential for the community acquired isolates to develop multidrug resistance in hospital environment and may pose a major challenge for clinical management.

## Background

*Klebsiella pneumoniae* is a common cause of nosocomial and community acquired infections especially in immunocompromised patients. *K. pneumoniae* is categorised into three phylogenetic groups: KpI, KpII and KpIII. KpI comprises of *K. pneumoniae* subsp. *pneumoniae*, *K. pneumoniae* subsp. *rhinoscleromatis* and *K. pneumoniae* subsp. *ozaenae*. Recently, KpII has been designated as *K. quasipneumoniae* and was further classified into KpIIa which is *K. quasipneumoniae* subsp. *quasipneumoniae* and KpIIb which is *K. quasipneumoniae* subsp. *similipneumoniae* [1]. The KpIII group comprises of *K. variicola*. Accurate phenotypic identification of these species is difficult and can be achieved by molecular techniques [1].

*K. pneumoniae* which is commonly associated with urinary tract infection, pneumonia, bacteraemia and wound infections, is also a primary causative agent of liver abscess particularly in Asia. The strains causing liver abscess are mostly community acquired and associated with a hypermucoid phenotype. These hypermucoviscous types, also called as hypervirulent (hv) *K. pneumoniae* (Kp) are often susceptible to antimicrobials [2]. They have the potential for metastatic spread in young and healthy individuals without a history of hepatobiliary disease [2]. In 2016, Breurec et al. reported *K. quasipneumoniae* subsp. *quasipneumoniae* with hypervirulent characters causing liver abscess [3]. Molecular identification of these strains is by the detection of *rmpA*, *rmpA2* and *magA* genes by PCR. *rmpA* and *rmpA2* are the regulators of mucoid phenotype; *rmpA2* shares 80% identity with 3’ DNA sequences of *rmpA* [4]. *magA*, codes for the K1 mucoviscous serotype. Though these markers are described, hypervirulent *K. pneumoniae* has no internationally agreed definition and different terms have been used by investigators. Some authors define hypervirulence by the presence of a mucoid phenotype whilst others define based on the detection of *rmpA* and *rmpA2*. Recently *iutA* gene encoding aerobactin has been used to define hvKp [5]. Other authors have considered virulence factors such as siderophores, type3 fimbriae and allantoin metabolism [6].

The objective of this study is to characterise the hypervirulent strains isolated from bacteraemic patients for (i) determination of various virulence genes and resistance genes, (ii) to investigate the difference between healthcare associated and community acquired hvKp isolates with respect to clinical profile, antibiogram, clinical outcome and molecular epidemiology.

## Methods

### Bacterial isolates

*K. pneumoniae* isolates from bacteremic patients during 2015 were collected at the department of Clinical Microbiology, Christian Medical College, India. The isolates were speciated using standard culture, biochemical and identification procedures [7]. String test was used to identify the hypervirulent phenotype. It was defined as positive if there was formation of mucous string of >5 mm when the bacterial colony was stretched, with an inoculation loop [2]. During the study period, twenty seven isolates were positive for string test. Among which, seven isolates with well-defined criteria for hypervirulence based on PCR positive for virulence genes *rmpA* and *rmpA2* and representative of community and hospital acquired bacteremia were chosen for further characterization. In this study, hypervirulent strains are defined as those that are string test positive and carry both *rmpA* and *rmpA2*.

### Antimicrobial susceptibility testing

Antimicrobial susceptibility testing was performed by Kirby Bauer disc diffusion method for the commonly used antimicrobials as described by CLSI [8]. The panel of antimicrobials tested includes ceftazidime (30 μg), cefepime (30 μg), piperacillin/tazobactam (100/10 μg), meropenem (10 μg), gentamicin (10 μg), amikacin (30 μg), ciprofloxacin (5 μg) and tigecycline (15 μg). *E. coli* ATCC 25922 was the control strain used for susceptibility testing. The results were interpreted according to CLSI 2015 guidelines [9]. For tigecycline, interpretation was according to FDA criteria (http://www.accessdata.fda.gov/drugsatfda_docs/label/2009/021821s016lbl.pdf).

### Molecular characterisation of hypervirulent *K. pneumoniae*

#### PCR for *rmpA*, *rmpA2* and capsule types 1&2

String test positive isolates were then subjected to conventional PCR for *rmpA*, *rmpA2*, *magA* and *K2* genes as described in previous studies [10, 11]. *magA* is used to identify K1 capsular type and *K2wzy* is for the K2 capsular type.

#### Whole genome sequencing (WGS) for hv *K. pneumoniae*

The seven isolates that were positive for *rmpA*, *rmpA2* and susceptible to all the antimicrobials tested, were subjected to whole genome sequencing. DNA was extracted using Qiasymphony (Qiagen) as per manufacturer’s instructions. The whole genome sequencing was done with Ion Torrent PGM platform using 400 bp read chemistry as described in our previous report [12]. Raw reads were assembled *de novo* using Assembler SPAdes software v5.0 in Torrent suite server version 4.4.3. Coverage of the genomes ranged from 6.5X to 87.6X for the seven isolates. Annotation of the genomes was performed with RAST (Rapid Annotation using Subsystems Technology- http://rast.nmpdr.org/) and PATRIC (Pathosystems Resource Integration Centre - https://www.patricbrc.org/). The whole genomes shotgun sequences were deposited at GenBank to obtain accession numbers.

The annotated whole genome sequences (WGS) were used for the prediction of antibiotic resistance genes, plasmids and the sequence type from the ResFinder, PlasmidFinder and MLST finder tool, respectively, of the Centre for Genomic Epidemiology (https://cge.cbs.dtu.dk/). The list of alleles for the virulence genes of *K. pneumoniae* was provided by the Pasteur Institute and was screened from the WGS data accordingly (http://bigsdb.pasteur.fr/perl/bigsdb/bigsdb.pl?db=pubmlst_klebsiella_seqdef_public&page=profiles).

The relatedness of the predicted sequence types were investigated by eBURST V3 software employing the BURST algorithm. Genetic relatedness and comparative genomic analysis of the isolates were performed using EDGAR software (https://edgar.computational.bio.uni-giessen.de/cgi-bin/edgar_login.cgi).

### Demographic and clinical details of the patients

The demographic and clinical details of the patients including age, sex, source of infection, co-morbidities and 30 day outcome were obtained from electronic medical records of the hospital. Healthcare associated infection was defined as blood culture positive in the first 48 h of admission in a patient hospitalized for at least two days within the last 90 days, receiving specific home nursing care or attending hospital or haemodialysis clinic in the 30 days before infection. Community acquired infection was defined as blood culture positive within 48 h of admission in a patient without prior hospitalization in last 90 days.

## Results

The clinical details of the seven patients are summarised in Table 1. The patients aged from 29 years to 88 years with median age of 55 years. Five of the seven patients were male. All patients had a co-morbid condition. Three patients had intra-abdominal infection but none had liver abscess or metastatic infection. At 30 days three patients expired. Three isolates were from patients with community acquired infection while four were from patients with healthcare associated infections. All three patients with community acquired infection had chronic liver disease and expired. The four patients with hospital acquired infection had no chronic liver disease and all survived.

**Table 1:** Demographic and clinical profile with co-morbidities of the patients with hvKp

Isolate number	Source of sepsis	CAI/HCA	30-day outcome	DM	CKD	CLD	Malignancy
Kp1	Line infection	HCA	Alive	Absent	Absent	Absent	Absent
Kp2	Pneumonia	HCA	Alive	Absent	Absent	Absent	Present
Kp3	Unclear	HCA	Alive	Absent	Present	Absent	Absent
Kp4	Intra-abdominal	CAI	Expired	Absent	Absent	Present	Absent
Kp5	Intra-abdominal	HCA	Alive	Absent	Absent	Absent	Present
Kp6	Intra-abdominal	CAI	Expired	Present	Absent	Present	Absent
Kqp	SSTI (Necrotising fasciitis)	CAI	Expired	Absent	Absent	Present	Absent

*Kp K. pneumoniae*, *Kqp K. quasipneumoniae*, *DM* Diabetes Mellitus, *CKD* Chronic Kidney Disease, *CLD* Chronic liver Disease, *CAI* Community acquired infection, *HCA* Healthcare associated infection, *SSTI* Skin and soft tissue infection

The seven isolates were susceptible to all the antimicrobials tested. The isolate Kp3 was resistant to ciprofloxacin. Though the isolates were phenotypically susceptible to all tested antimicrobials, six harboured *oqxA* and *oqxB* genes coding for fluoroquinolone resistance. *bla*_SHV_ gene is intrinsic to *K. pneumoniae* and the variants *bla*_SHV-36_ and *bla*_SHV-75_ were detected in the study isolates. As per annotation from NCBI, one isolate was found to be *K. quasipneumoniae* subsp. *similipneumoniae* and belonged to K1 capsular type. *K. quasipneumoniae* carried the *bla*_*OPKB*-2_ gene which is inherently present in this species and can serve as a marker for identification (Breurec et al., 2016).

Results of PCR for *magA* (K1) and *K2wzy* genes are summarised in Table 2 along with the accession number of the genomes. Molecular determination of capsule revealed four isolates belonged to K1 capsular type indicated by the presence of *magA* gene. Three isolates did not belong to K1 or K2 capsular types by PCR. However, from the whole genome sequence analysis, Kp1 and Kp5 were found to belong to K20 and K1 respectively. Isolate Kp6 was untyped in the study.

**Table 2:** Results of the whole genome analysis showing resistance genes, virulence genes, plasmid profile, MLST and capsule typing by PCR

Isolate number	Accession number of genomes	*magA*/K1 (PCR)	*K2wzy*/ K2 (PCR)	Sequence type (ST)	Resistance genes	Plasmids	Fimbriae	Iron uptake	Allantoin metabolism	Siderophores	Others
Kp1	LZSB00000000	Negative	Negative	420	*oqxA* and *oqxB*	IncHI1B, IncFIB	*mrkD*	*fyuA*	–	*entB, entF, iroN, iroB, iroC, iutA, irp1, irp2, ybtA, ybtE, ybtS, ybtT*	MviM
Kp2	MLCD00000000	Positive	Negative	23	*bla*_*SHV-36*_, *oqxA* and *oqxB*	IncHI1B	*mrkD*	*kfuB, fyuA*	*allB, allR, allS, ybbW*	*entB, iutA, iroB, iroN, irp1, irp2, iucD, iucC, ybtE, ybtT, ybtP, ybtA, ybtU*	MviM
Kp3	MBQC00000000	Positive	Negative	23	*bla*_*SHV-36*_, *oqxA* and *oqxB*	IncHI1B	*mrkD*	*kfuA, kfuB, kfuC*	*allB, allR, allS, ybbW*	*entB, iutA, iroN, iucA, iucB, iucC, iucD, irp1, ybtE, ybtP, ybtU,*	MviM
Kp4	MBFY00000000	Positive	Negative	23	*bla*_*SHV-36*_, *oqxA* and *oqxB*	IncFIIK, IncHI1B and IncR	*mrkD, mrkB*	*kfuA, kfuB, kfuC*	*allB, allR, allS, ybbW*	*entB, entF, clbK, clbN, iroB, iroC, iroD, iroN, iutA, iucA, iucB, iucC, iucD, ybtU, ybtP*	mceC, mceD
Kp5	LZPA00000000	Positive	Negative	23	*bla*_*SHV-36*_, *oqxA* and *oqxB*	IncHI1B	*mrkD*	*kfuA, kfuB, kfuC, fyuA*	*allB, allR, allS*	*entB, iutA, ColicinV, irp2, iutA, iucA, iucB, iucC, iucD, ybtE*	MviM
Kp6	MBQB00000000	Negative	Negative	2319	*bla*_*SHV-75*_ and *oqxA*	IncHI1B	*mrkC, mrkD*	*fyuA*	–	*entB, iroB, iroC, iroD, iroN, iutA, irp1, irp2, iucC, ybtE, ybtT, ybtU*	MviM
Kqp	MBSL00000000	Positive	Negative	2320	*bla*_*OKPB 2*_	IncHI1B	*mrkD*	*–*	*–*	*entB, iroB, iroN, iutA, iucB, iucD, irp1, irp2, ybtA, ybtE, ybtQ, ybtU, ybtT*	MviM

*Kp K. pneumoniae*, *Kqp K. quasipneumoniae*

The whole genome sequences were analysed for virulence genes and are listed in Table 2. Ferric iron uptake was encoded by genes including *kfuA*, *kfuB* and *kfuC* (ferric iron ABC transporter). The *fyuA* gene, which encodes an outer membrane receptor for ferric siderophores, was also present. Allantoin metabolism was controlled by genes *allB (*allantoinase), *allR* (negative regulator), *allS* (transcriptional activator) and *ybbW* (allantoin permease). Genes encoding enterobactin identified in the study isolates were *entB* (isochromatase) and *entF* (enterobactin synthetase component). Salmochelin, a derivative of enterobactin, was encoded by *iroB* (glycosyltransferase), *iroC* (ABC transporter protein) and *iroN* (outer membrane siderophore receptor). Genes coding for aerobactin identified in the study were *iutA* (aerobactin siderophores receptor), *iucA* (N(2)-citryl-N(6)-acetyl-N(6)-hydroxylysine synthase), *iucB* (N6-hydroxylysine O-acetyltransferase), *iucC* (Aerobactin synthase) and *iucD* (L-lysine 6-monooxygenase). Yersiniabactin encoding genes identified in the genomes were *irp1* (polyketide synthetase), *irp2* (iron acquisition yersiniabactin synthesis enzyme), *ybtA* (iron acquisition regulator), *ybtE* (2,3-dihydrobenzoate-AMP ligase), *ybtP* (putative inner membrane ABC transporter), *ybtQ* (putative ABC iron siderophore transporter), *ybtT* (yersiniabactin synthesis enzyme) and *ybtU* (thiazolinyl imide reductase)*.*

The whole genome analysis revealed the presence of plasmids in all seven isolates. All isolates harboured the plasmid IncHI1B but did not carry any resistance genes. IncR and IncFIB were found in one isolate each. Six isolates harboured the plasmid pLVKP which is characteristically associated with hypervirulent strains. The plasmid is known to contain *rmpA, rmpA2* and *iutA* genes. This plasmid could not be located in Kp2, although it contained the *rmpA* and *rmpA2* genes, which can be attributed to missing sequences during the contig arrangement of the raw reads in Ion Torrent. All the isolates harboured *mrkD* which aids in biofilm formation. All the *K. pneumoniae* isolates coded for genes responsible for allantoin metabolism which the *K. quasipneumoniae* lacked.

Among the various sequence types seen, *magA* gene (K1 capsular type) was seen among the isolates belonging to ST23 which is characteristic to this clonal type. One isolate belonged to ST420 and two isolates had novel sequence types namely ST2319 and ST2320. The novel sequence types submitted to Institut Pasteur MLST system (Paris, France) and were assigned new sequence types. *K. quasipneumoniae* belonged to the novel sequence type ST2320 and K1 capsular type.

**Fig. 1:**
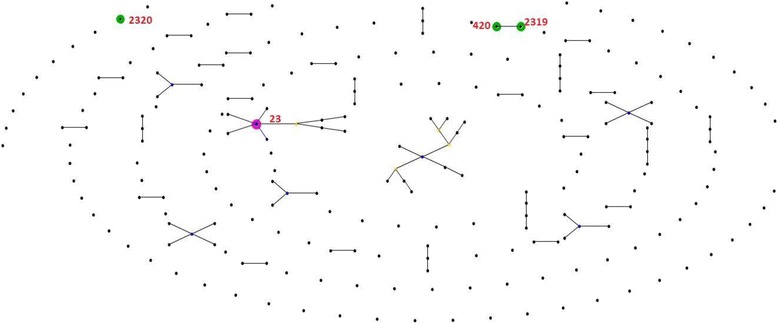
eBURST analysis of seven hypervirulent *Klebsiella pneumoniae*

eBURST analysis of the seven isolates is shown in Fig. 1. The sequence types seen in the study isolates form separate clusters as shown. However, ST2319 which is a newly assigned clonal type is a single locus variant of ST420. The two STs differ in the tonB allele with ST420 having tonB-36 and ST2319 having tonB-176. Hence these two clonal types are closely related.

**Fig. 2:**
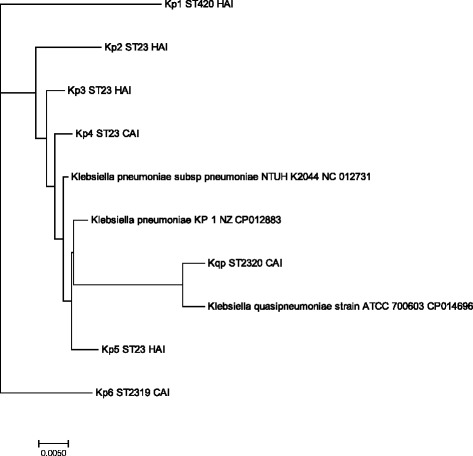
Phylogenetic tree of the hypervirulent *K. pneumoniae.* The phylogenetic tree for 10 genomes built out of a core of 2999 genes per genome and 29,990 in total. The core has 958,416 AA-residues/bp per genome, 9,584,160 in total. Each entry is represented by isolate name followed by sequence type and type of infection. HAI: Healthcare associated infection, CAI: Community acquired infection, ST: Sequence type

Figure 2 shows the phylogenetic tree of the study isolates which was constructed using EDGAR software with three reference genomes namely *K. pneumoniae* subsp. *pneumoniae* NTUH K2044 NC 012731, *K. pneumoniae* KP1 NZ CP012883 and *K. quasipneumoniae* ATCC 700603 CP014696. The *K. quasipneumoniae* isolate in the present study is related to the *K. quasipneumoniae* ATCC700603 isolate. Other isolates such as Kp2, Kp3, Kp4 and Kp5 are closely related to the reference genomes used while KP1 and Kp6 stand out as separate groups. From Fig. 2, the result of phylogenetic tree shows that the ST23 are closely related while the ST420 and ST2319 are more diverse.

**Fig. 3:**
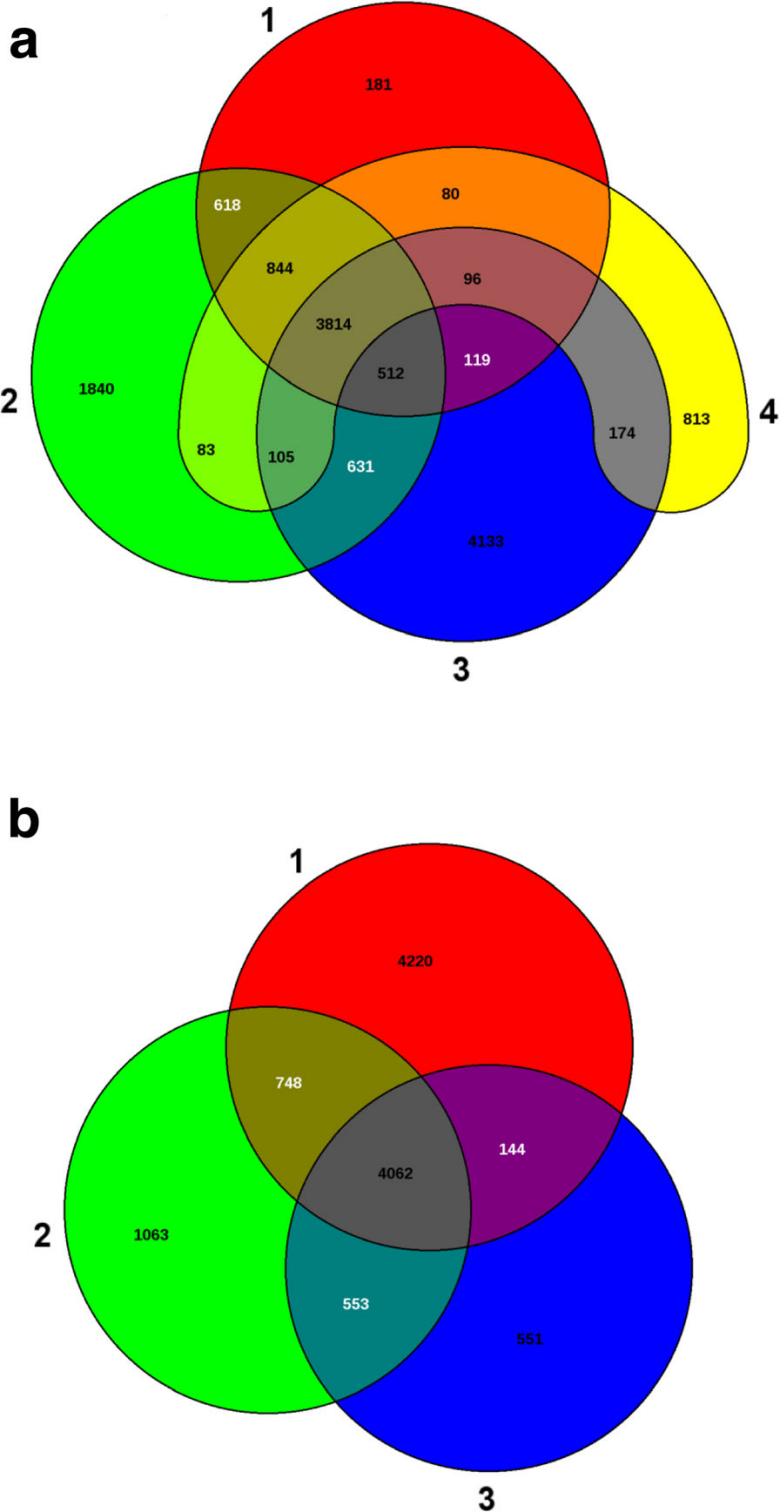
**a:** Venn diagram of the four healthcare associated hypervirulent *K. pneumoniae* isolates. Isolate1: Kp3, isolate2: Kp2, isolate3: Kp1 and isolate4: Kp5. Kp3 and Kp5 have 181 and 813 genes respectively exclusive to the isolates and share a lot of genes with other two genomes. **b:** Venn diagram of community acquired *K. pneumonia* and *K. quasipneumoniae* isolates. Isolate1: Kp6, isolate2: Kp4 and isolate3: Kqp. Kp6 has 4220genes exclusive to the isolate and the community acquired isolates share lesser genes among themselves than the healthcare acquired isolates. *K. quasipneumoniae* is a smaller genome when compared to the *K. pneumoniae* isolates

Figure 3a depicts the comparative Venn diagram for the four healthcare associated hypervirulent isolates showing the genes shared by them. Kp3 and Kp5 have 181 and 813 genes respectively exclusive to the isolates and share a lot of genes with other two genomes. Figure 3b is the venn diagram of the three community acquired hypervirulent isolates. Kp6 has 4220genes exclusive to the isolate and the community acquired isolates share lesser genes among themselves than the healthcare acquired isolates. *K. quasipneumoniae* is a smaller genome when compared to the *K. pneumoniae* isolates.

## Discussion

This case series is the first report from India characterizing hvKp isolates from bacteremia using WGS. There have been genome announcements of hypervirulent strains [13–15] but there have been no reports from India on molecular analysis of the same enumerating the virulence genes. Most studies describe hvKp in isolates from liver abscesses; studies on hvKp from patients with bacteraemia are limited and lacking from the Indian setting. Seven susceptible isolates were chosen for characterisation since classically hvKp were susceptible to all antimicrobials. They have been associated more with community acquired infections such as liver abscess than healthcare associated infections. Interestingly, all three patients with community acquired infection in this study had underlying liver disease and succumbed to infection. This may be due to late presentation to hospital when these patients were critically ill. Community acquired hvKp infections are often associated with high mortality rates [2]. In contrast, no patients with hospital acquired infection had underlying liver disease and all survived.

Though the isolates harboured β-lactamase genes such as *bla*_*SHV-36*_ and *bla*_*SHV-75*_*,* they were phenotypically susceptible to β-lactam antibiotics. *bla*_*SHV*_ contributes to the intrinsic resistance to ampicillin in *K. pneumoniae* [16]*.* Genes encoding resistance to fluoroquinolones such *oqxA* and *oqxB* were present in all but only one strain was found resistant to ciprofloxacin by disc diffusion. All the isolates harboured the IncHI1B type of plasmid. However, there was no resistance gene found associated with this plasmid. The hvKp strains have been characteristically susceptible to antibiotics although recent reports suggest the emergence of MDR-hvKp [17–19].

*K. pneumoniae* has several virulence factors which includes the ability to produce siderophores, such as aerobactin, enterobactin and yersiniabactin, genes involved in ferric iron uptake, allantoin metabolism and biofilm formation [2]. Strains of *K. pneumoniae* capable of over-producing siderophores are considered to be hypervirulent [20, 21]. The strains which do not secrete siderophores have decreased virulence and hence are less efficient at infection and colonization. Sah et al.*,* demonstrated that siderophores are important for pathogenic *K. pneumoniae* to survive in low iron concentration [22]. Pomakova et al.*,* showed that hvKp was able to survive longer in rat abscess model as well as in 90% human serum than the classical Kp strains [23]. The hypermucoviscous nature and added virulence factors contribute to the increased severity of infections caused by hvKp, despite susceptibility to first and second line antibiotics.

The hvKp in this study was defined based on the clinical features, positivity to string test and presence of *rmpA* and *rmpA2*. Seven isolates were chosen for WGS by Ion Torrent to characterise the virulence genes. The plasmid pLVPK (Large Virulence Plasmid of *K. pneumoniae*) was identified. This plasmid harbours the *rmpA*, *rmpA2* genes and also the multiple iron acquisition systems *iucABCDiutA* and *iroBCDN* siderophore gene clusters [24]. These genes were identified in the isolates harbouring the pLVPK in this study. An in vivo study in a mouse model shows the absence of dissemination of hvKp from liver abscess when there is loss of pLVPK [25]. Jung et al., also observed the presence of *rmpA2* in all the *rmpA* positive isolates similar to our study findings [21].

Several genes coding for aerobactin, yersiniabactin and their receptors were present in all the seven study isolates as listed in Table 2. A previous report by Hsieh et al., reports that these siderophores were more frequent in hvKp strains than the cKp strains [26]. Jung and colleagues in Korea reported the presence of aerobactin gene in all the study isolates and the gene for allantoin metabolism, *allS,* was present in 50% of the hvKp isolates [21]. Aerobactin enables the survival of hvKp in vivo and ex vivo and plays a significant role than the other siderophores such as enterobactin and yersiniabactin [27]. *ybtQ*, coding for a transporter for yersiniabactin was present in *K. quasipneumoniae.* This has been demonstrated to have an important role for the survival of *K. pneumoniae* in iron limiting condition [28].

Genes for allantoin metabolism were present in 57% of the study isolates comparable to the findings by Jung et al. [21]. The genes *rmpA*, *rmpA2*, aerobactin and *allS* were found frequently in hvKp isolates than the classical Kp (cKp) isolates from bacteremia [21]. All the isolates in this study harboured *mrkD* which is an adhesin and aids attachment to acellular surfaces. One isolate each showed the presence of *mrkB*, chaperone, and *mrkC*, an outer membrane usher gene [29]. These genes are essential in pili formation and are important for biofilm formation and colonisation. To the best of our knowledge this is the first study to report the presence of *mrkB* and *mrkC*. All the isolates had the gene MviM which is a virulence factor but the significance has not been described till date.

Microcins are low molecular weight antimicrobial peptides produced by *Enterobacteriaceae* against other gram negative bacteria. In one isolate, *mceC* and *mceD* genes were identified. *mceC* is involved in the maturation of microcin while the function of *mceD* has not been defined [30].

When investigating isolates from patients with liver abscess, Yu et al. found the hypermucoviscous phenotype, *rmpA*, aerobactin, *kfu* and *allS* were present in 96%, 100%, 100%, 100%, and 100% respectively of K1 type isolates; whereas in nonK1/K2 isolates, they were present in 79%, 86%, 93%, 50% and 0% respectively [31]. This highlights the association between virulence genes and capsular type in hvKp. In the present study, four isolates of K1 type had a similar virulence gene profile while the *K. quasipneumoniae,* although of K1 type, lacked genes for iron uptake and allantoin metabolism. This can also be attributed to clonal types of the isolates. Four isolates of K1 capsular type belonged to ST23. Kp6 belonging to ST2319 is closely related to ST420 are shown in Fig. 1 and both do not belong to capsular type K1 or K2. *K. quasipneumoniae* belonging to K1 capsular type and ST2320 is an outgroup.

HvKp are frequently associated with ST23 and capsular types K1 or K2 [32, 33]. In this study, four isolates belonged to ST23, all of which were K1 type. Kp1 belonged to K20 which is also associated with hvKp [2] and Kp6 was untyped. The isolates belonging to ST23 had a similar virulence profile. The isolate belonging to ST420 differed from the isolates belonging to ST23 in lacking genes for allantoin metabolism. Similar result was observed with novel types ST2319 and ST2320. The presence of large number of virulence genes could explain the high pathogenicity reported in ST23 isolates than the other clonal types. The presence of the international circulating clone in India may threaten the management of infections with MDR *K. pneumoniae*. ST420 with *rmpA* has previously been reported in a patient with a liver abscess; similar to the isolate in this study patient [34]. Three patients with hospital acquired infection had ST23; these patients were on different wards at different times which suggest that this was not part of an outbreak. Community acquired hvKp infections of ST23 have been commonly reported but here ST23 is associated with hospital infection. Hence containment of hvKp infections is essential which requires monitoring the incidence of these infections. One of the two new sequence types, ST2319 is a variant of ST420 differing in the *tonB* allele. This indicates the emergence of strains with new sequence types and virulence characteristics.

## Conclusion

The concerns of hvKp include its prevalence among bacteraemic isolates without the presence of liver abscess. The hypervirulent strains are now being reported not only among *K. pneumoniae* but also among *K. quasipneumoniae*. To date, predominant sequence types associated with hvKp include ST23 and ST57. Here we report new clonal types among hvKp, ST2319 and ST2320, which indicates that these strains are spreading and no longer restricted to selected clonal groups. This is of concern as these strains confer more severe disease with higher mortality rates. It is also concerning that four out of seven of our isolates were from patients with healthcare associated infection whereas previously hvKP has been seen mainly in the community. The community acquired strains as in the present study lead to fatal outcome unlike nosocomial strains. Also, there is potential for the community acquired hypervirulent isolates to develop multidrug resistance in the hospital environment and may become difficult to manage. Further studies are required to monitor their occurrence and antimicrobial susceptibility profile. Also, there is urgent need to establish an international definition and definitive identification markers for hypervirulent *K. pneumoniae*.

## Abbreviations

*allB*: allantoinase
*allR*: negative regulator for allantoinase
*allS*: transcriptional activator
ATCC: American Type Culture Collection
CLSI: Clinical Laboratory Standards Institute
*entF*: enterobactin synthetase component
FDA: Food and Drug Administration
hvKp: hypervirulent *Klebsiella pneumoniae*
Inc.: Incompatible
*iroB*: glycosyltransferase
*iroC*: ABC transporter protein
*iroN*: outer membrane siderophore receptor
*irp1*: polyketide synthetase
*irp2*: iron acquisition yersiniabactin synthesis enzyme
*iucA*: N(2)-citryl-N(6)-acetyl-N(6)-hydroxylysine synthase
*iucB*: N6-hydroxylysine O-acetyltransferase
*iucC*: Aerobactin synthase
*iucD*: L-lysine 6-monooxygenase
*iutA*: aerobactin siderophores receptor
Kp: *Klebsiella pneumoniae*
Kqp: *Klebsiella quasipneumoniae*
*magA*: mucoviscosity associated gene
*mce*: Microcin
*mrkD*: type 3 fimbriae
PATRIC: Pathosystems Resource Integration Centre
PCR: Polymerase Chain Reaction
pLVKP: Large Virulence plasmid of *Klebsiella pneumoniae*
RAST: Rapid Annotation using Subsystems Technology
*rmpA*: regulator of mucoid phenotype
ST: Sequence type
WGS: Whole genome sequencing
*ybbW*: allantoin permease
Ybt: Yersiniabactin
*ybtA*: iron acquisition regulator
*ybtE*: 2,3-dihydrobenzoate-AMP ligase
*ybtP*: putative inner membrane ABC transporter
*ybtQ*: putative ABC iron siderophore transporter
*ybtT*: yersiniabactin synthesis enzyme
*ybtU*: thiazolinyl imide reductase
